# Quantifying pigment cover to assess variation in animal colouration

**DOI:** 10.1093/biomethods/bpx003

**Published:** 2017-03-27

**Authors:** Andjin Siegenthaler, Debapriya Mondal, Chiara Benvenuto

**Affiliations:** School of Environment and Life Sciences, University of Salford, Salford M5 4WT, UK

**Keywords:** chromatophores, chromatosomes, Chromatophore Index, colour change, colour threshold, *Crangon crangon*, ImageJ

## Abstract

The study of animal colouration addresses fundamental and applied aspects relevant to a wide range of fields, including behavioural ecology, environmental adaptation and visual ecology. Although a variety of methods are available to measure animal colours, only few focus on chromatophores (specialized cells containing pigments) and pigment migration. Here, we illustrate a freely available and user-friendly method to quantify pigment cover (PiC) with high precision and low effort using digital images, where the foreground (i.e. pigments in chromatophores) can be detected and separated from the background. Images of the brown shrimp, *Crangon crangon*, were used to compare PiC with the traditional Chromatophore Index (CI). Results indicate that PiC outcompetes CI for pigment detection and transparency measures in terms of speed, accuracy and precision. The proposed methodology provides researchers with a useful tool to answer essential physiological, behavioural and evolutionary questions on animal colouration in a wide range of species.

## Introduction

The study of animal colouration and colour patterns is essential to gather a better understanding on how animals visually communicate and how they can match different substrates. Furthermore, this type of studies provides important insights on how predation avoidance due to camouflage can drive inter- and intraspecific variation, and how colouration and visual perception are connected (e.g. [1]). A wide range of methods has been developed to measure animal colouration, which can be roughly divided in three categories: (i) spectral quantification of colouration and animal vision [2, 3]; (ii) assessment of colour patterns [4–7]; and (iii) analysis of chromatophores and pigment migration [8–10]. The last method has been used mainly to study animal colour changes [9, 11, 12].

Chromatophores are specialized cells containing pigmented organelles and can be located in the dermis, epidermis, beneath a translucent exoskeleton, deep in muscular tissue or around internal organs [13–15]. In crustaceans, multiple tightly bound chromatophores (of similar or different colours) are combined in a structure called chromatosome [13, 16]. Many animals can regulate their colour by the dispersal and concentration of pigments within chromatophores (e.g. [12, 17]): colour can be changed in a period of days to months through anabolism and catabolism of pigments and cells (morphological colour change) or within milliseconds to hours via the migration of pigments within chromatophores (physiological colour change) [12]. The concentration or dispersion of pigments reduces or increases their visibility, since less or more surface area is covered by them, respectively [18, 19]. Hogben and Slome [20] described changes in the pigment distribution in the frog *Xenopus laevis* by classifying chromatophores in five classes (Supplementary Fig. S1), applying a Melanophore Index (MI) for melanophores (also more generally called Chromatophore Index (CI) for chromatophores containing pigments other than melanin [20, 21]). Although this method has been extensively used (see Table 1 for some recent examples), concerns have been raised about its degree of subjectivity, statistical validity and labour intensiveness [22, 23]. Here, we describe a new method, PiC (Pigment Cover), to assess the degree of pigment dispersion within chromatophores (or chromatosomes) by measuring the coverage of pigments in defined areas of an animal body, thus allowing us to evaluate colour variations in a quantitative way. The objective of this study is to demonstrate the use and versatility of PiC and compare it to the established CI. To achieve this, both PiC and CI were applied to a database of pictures of the brown shrimp, *Crangon crangon* (L.), a crustacean characterized by good background-matching abilities [8].

**Table 1:** Selected publications applying the MI of Hogben and Slome [20]

Group	Species	Area of interest	Topic	Method	Source
Amphibian	*Bufo melanostictus*	Dorsal skin	Drug development	MI^a^	[24]
	*Hoplobatrachus tigerinus*	Isolated dorsal skin cell	Physiology	MI^a^	[25]
	*Rana catesbeiana*	Dorsal skin	Endocrinology	MI	[26]
	*Taricha granulosa*	Larva	UV protection	MI	[27]
	*Ambystoma gracile*	Larva	UV protection	MI	[27]
	*Ambystoma macrodactylum*	Larva	UV protection	MI	[27]
	*Xenopus laevis*	Larva	Developmental biology	MI	[28, 29]
Crustacean	*Chasmagnathus granulata*	Maxilliped’s meropodit	UV protection	CI	[30]
	*Palaemonetes argentinus*	Dorsal abdomen	UV protection	CI	[30]
	*Eurydice pulchra*	Not specified	Endocrinology	CI^a^	[31]
	*Palaemon pacificus*	Dorsal abdomen	Endocrinology	CI	[32, 33]
Reptile	*Hemidactylus flaviviridis*	Dorsal skin	Drug development	MI^a^	[34]
Teleost	*Ctenopharyngodon idellus*	Scale	Physiology	MI	[35]
	*Danio rerio*	Scale and embryo	Physiology	MI	[36]
	*Oncorhynchus mykiss*	Scale	Ecotoxicology	MI	[37]
	*Verasper moseri*	Base of caudal fin	Developmental biology	MI	[38]

^a^Modified index; MI, Melanophore Index (pigment is melanin); CI, Chromatophore Index (pigment is not melanin).

## Material and methods

### Protocol to measure PiC 

#### Image acquisition

**Figure 1:**
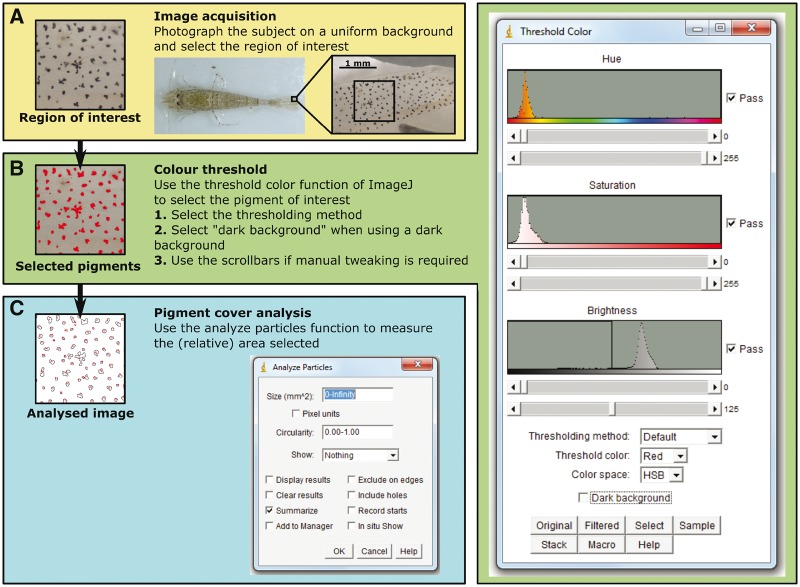
Protocol for PiC measurements. This diagram outlines the steps to be performed in ImageJ to determine PiC. See text for details.

Measurements on animal colour or pigment migration are usually performed on a specific body region rather than the whole animal [1, 10, 39]. In some cases, e.g. fish scales [36], the area of interest can be separated from the animal prior to image acquisition, reducing the effects of animal stress on the colour [40]. The specimen should be placed and photographed on a uniform surface (Fig. 1A). Contrast between background and pigments should be as high as possible; overlap with underlying organs should be avoided, if possible [23]. The magnification should be high enough to distinguish individual chromatosomes. If multiple pigments are studied, the collection of multiple images of the same area on different backgrounds might be necessary (see below). To optimize image acquisition, illumination within an image should be uniform and shadows or reflection of light should be avoided. Light conditions are, nevertheless, less constricted than in other methods (e.g. [3, 41]) and colour charts are not required (they can vary in quality and applicability; [2, 3]). Still, standardization of lighting conditions and camera settings will significantly reduce the use of manual adaptations during image analysis (see [41] for more information on the standardization of digital images). In digital photography, images are commonly displayed in a non-linear standard default colour space (sRGB). PiC can be applied to these standard images. For more rigorous and objective image analyses, linear images are often required. If this is the case, sRGB images can be converted to the CIELAB colour space using the ‘Color Space Converter’ plugin of ImageJ (https://imagej.nih.gov/ij/plugins/color-space-converter.html). A normalization step is advised to slightly enhance the contrast within the images by using the ‘Enhance Contrast’ command of ImageJ. A slight over-saturation of 1% is advised for improved visual evaluation [48].

#### Colour threshold

PiC image analysis can be performed with any graphic editor able to perform image segmentation (partitioning an image into sets of pixels) by means of thresholding. Image segmentation by semi-automatic thresholding is an established method that has been used in a range of biological studies, including crop root length [42], plant signals [43] and cell counts [44], but not specifically on pigment coverage. The methodology described in this section is tailored to the freely available java-based imaging program ImageJ (1.48v, http://imagej.nih.gov/ij/; [45]; RRID:SCR_003070) because of its ease of use and efficacy, but could easily be adapted to other graphic software.

Images need to be cropped to the region of interest and segmented to differentiate foreground (the pigments under study) and background [46]. In ImageJ, sRGB image segmentation is achieved with the ‘Color Threshold’ function (Fig. 1B), which segments 24-bit RGB images based on pixel values (see the ImageJ user guide; [47]). A range of automatic thresholding algorithms is available in ImageJ. These algorithms perform differently depending on the distribution of pixel values in the image and the most suitable thresholding algorithm should be selected prior to analysis [42, 48], e.g. using the ‘Threshold Check’ macro of the BioVoxxel toolbox (http://www.biovoxxel.de/development/, http://fiji.sc/BioVoxxel_Toolbox#Threshold_Check). The sensitivity of the threshold function can be manually adapted using the ‘Saturation’ and ‘Brightness’ scroll bar in the colour threshold settings window (Fig. 1B) until the whole area covered by the pigment(s) of interest is selected [44, 49]. Manual alteration of the thresholding level reduces, however, the objectivity of the analysis and should be avoided as much as possible. Specific pigments can also be selected by adapting the ‘Hue’ scroll bar (Fig. 1B) to the required hue values [43]. For transparency measurements, the ‘Hue’ scroll bar should be used to select the background colour to ensure that only the transparent area is selected (the background will be visible through the transparent tissue) and all pigments are ignored. In cases where only one channel of the image is analysed (e.g. CIELAB’s L channel or greyscale images), ImageJ’s ‘Threshold’ function can be used in similar fashion as the ‘Color Threshold’ function.

#### PiC analysis

The area of the selected pigment(s) can be calculated with the ‘Analyze Particles’ command (Fig. 1C) that measures ‘particles’ (separate shaped objects) in an image after thresholding by scanning the image and outlining the edge of objects found has been performed [47, 50].

### Case study

#### Dark and light pigment measurements and transparency

**Figure 2:**
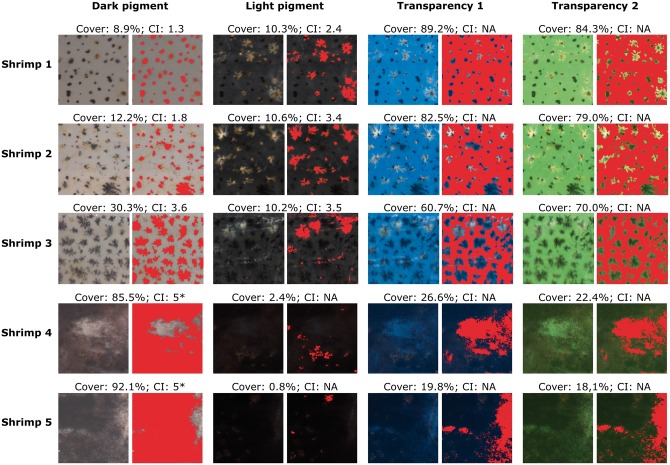
Pigment and transparency values for cover (%) and CI for five shrimps (*C. crangon*) on different backgrounds. For each specimen, the right exopod was photographed, always in the same exact position and then the image cropped in the centre (selecting 1 mm^2^). Red areas represent the area selected by the PiC method. NA: CI cannot be calculated. *CI is an estimate.

Five specimens of *C. crangon* were selected based on visual differences in colour. Their right exopod (the external branch of their tail fan) was photographed under a stereo microscope (Leica S6D) with a Leica DFC295 camera. The tail fan is the most suitable body area of caridean shrimp to be used for monitoring chromatic parameters because: (i) it is very flat; (ii) it has no underlying organs or tissue (and is thus highly transparent); and (iii) it can be photographed while causing minimal stress to the animal [23, 51]. Artificial illumination was provided by two led spotlights (JANSJÖ; 88 lm; 3000 Kelvin) positioned at either side of the microscope. We adjusted the white balance prior to image collection and allowed the exposure time to be automatically adapted. Images were collected in sequence, on four differently coloured backgrounds (Fig. 2): white for the measurement of dark-coloured (black and sepia-brown) pigments; black for light-coloured (white and yellow) pigments and green and blue for transparency measurements. Green and blue hues do not occur naturally in *C. crangon* [8, 51] and are, therefore, suitable for transparency measurements (both colours were used in order to test which one performs better). To avoid adaptation to the background during the measurements, shrimp were kept for a very short duration only (less than 1 min) on each background. Images were saved in uncompressed TIFF format (RGB), cropped to 1 mm^2^ and analysed following the protocol described above, using the default thresholding method, based on the IsoData algorithm [52, 53], and manually adapted if needed. We selected the default thresholding algorithm for this experiment since it performed best for the variety of features (dark pigment, light pigment, transparency) tested. For the same photos, we determined the CI, in accordance to the method of Hogben and Slome [20], by classifying all chromatosomes in the selected area individually and averaging their values (see Supplementary Fig. S1 for reference).

#### Dark PiC and CI comparison

**Figure 3:**
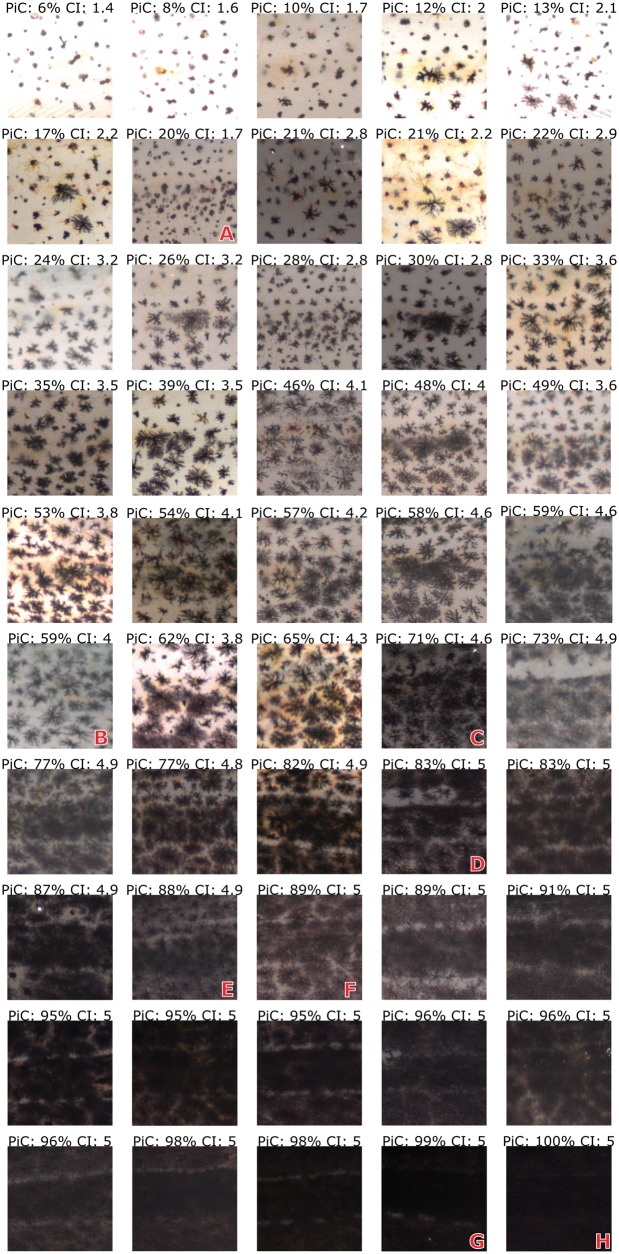
Percentage dark PiC and CI of 50 images (1 mm^2^) of *C. crangon*’s exopods. The images show different levels of chromatosome dispersion and represent the range of colouration exhibited by the animals. See text for information on capital letters.

Fifty sRGB images of *C. crangon* (Fig. 3; obtained from 36 individual shrimp) were selected to represent the range of colouration shown by shrimp (lighter or darker, depending on the substrate where animals were kept). We tested the robustness of the methodology used by selecting images varying in properties such as illumination and picture quality. All images were obtained on a white background and cropped to 1 mm^2^ in the centre of the exopod. Images were analysed for dark pigments, which are the most abundant and evident pigments responsible for dark colouration [8, 51]. Three observers analysed the images with the PiC and CI methods, in random order. Prior to analysis, we applied a threshold check (BioVoxxel toolbox) to a sub-selection of 13 images to determine the optimal thresholding algorithm. Based on the average score of these images, the MaxEntropy algorithm [54] was selected for all images. Manual adaptation was applied as little as possible (on average on 23% of the images, depending on the observer). To test the effect of image linearization and normalization, the 50 sRGB images were transformed to the CIELAB colour space and the L channel was normalized prior to PiC determination. The MaxEntropy thresholding algorithm was applied and, in this case, no manual adaptation was allowed to eliminate the need for subjective input. PiC values of the sRGB (averaged over the observers) and linearized/normalized images were compared using linear regression.

### Data analyses

Inter-observer variation for both dark PiC and CI was tested with the Friedman’s test. This statistical test was selected because of the non-normal distributed nature of both proportions and ordinal data, and the fact that each image was tested repeatedly. Both PiC (percentage of PiC transformed to fraction) and CI results were averaged between observers and a beta regression (betareg R package; [55]) was used to compare the methods. This specific analysis can also be important to predict the results from one method (PiC) when having information from the other (CI). Different link functions (log, log–log and logit) were compared based on Akaike Information Criterion (AIC). Beta regression is considered a suitable test for non-parametric and bounded data such as proportions [55]. Data analyses were performed with R statistical software v.3.1.2 (RRID:SCR_001905) and IBM SPSS statistics v. 20 (RRID:SCR_002865).

## Results

### Dark and light pigment measurements and transparency

For the five specimens analysed, dark PiC values ranged from 8.9% to 92.1% and light PiC values from 0.8% to 10.6% (Fig. 2). Transparency measurement ranged from 19.8% to 89.2% on a blue background and from 18.1% to 84.3% on a green background (mean difference ± SD: 1 ± 5.9%) and did not significantly differ between the background colours (Wilcoxon signed-ranks test: *N* = 5, *Z* = −0.674, *P* = 0.500). CI, by definition, cannot be calculated for transparency (Fig. 2). When dark pigments were predominant (e.g. shrimps 4 and 5 in Fig. 2), the CI of light pigments could not be calculated, as it was impossible to distinguish the chromatosomes' shape. Furthermore, the high overlap of dark chromatosomes made it impossible to count the number of chromatosomes to calculate the mean dark CI. In these cases, the CI was estimated as 5, the maximum index value.

### Dark PiC and CI comparison

**Figure 4:**
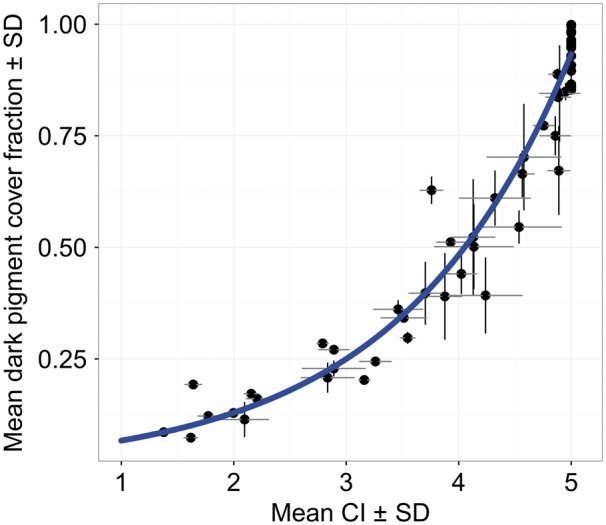
Relationship between CI and dark PiC fraction. Measurements were performed on 50 images of *C. crangon* (Fig. 3). Mean values and SD for the readings of three observers are given per image. The solid line shows the beta regression fit (with log link function).

PiC and CI for all 50 images were calculated (Fig. 3). Dark PiC showed a strong exponential relationship with CI (Fig. 4) and the beta regression confirmed a significant relationship between PiC and CI (coefficient ± SEM: 0.659 ± 0.034; *P* < 0.0001) with a pseudo-*R*^2^ value of 0.95. The equation to estimate PiC from a known CI value was modelled as:

Ln (predicted PiC)=−3.362 + 0.659*CI

The equation is only valid for: 1 ≤ CI ≤ 5 and 0 ≤ PiC ≤ 1. According to AIC values, the log link function (AIC: −127) provided a better fit than models with a logit (AIC: −82) or log–log (AIC: −66) link function.

In half of the images, the observers were not able to provide a reliable count of the maximum dispersed chromatosomes, necessary to calculate the CI, due to a high level of overlap between the chromatosomes. Above 63 ± 9% PiC, individual chromatosomes overlapped resulting in unreliable CI estimates; above 80 ± 9% PiC it was not possible to detect any difference based on CI since all chromatosomes were in the highest category (CI = 5). No problems were encountered during the estimation of PiC, including the darkest images. The observers spent on average 75 ± 5 min calculating the CI and only 18 ± 9 min determining PiC. Results differed significantly among observers for both methods (Friedman’s test: CI: *N* = 50, df = 2, *χ*^2^ = 11.09, *P* = 0.04; PiC: *N* = 50, df = 2, *χ*^2^ = 18.67, *P* < 0.001), with an average relative standard deviation over all images of 3% for CI and 6% for PiC. Individual regression parameters were similar among the observers (Supplementary Table S1) and the majority of the variation in the PiC estimates was caused by one observer who relied on manual adaptation (*N* = 23) much more than the other observers (*N* = 6 and *N* = 5). Linear regression estimates of PiC values of sRGB versus linearized/normalized images showed that both methods produce concordant results (Supplementary Fig. S2; df = 45, *R*^2^ = 0.995, *P* < 0.001, slope = 0.948), indicating that the use of sRGB images did not produce significant systematic errors in this case.

## Discussion

Animal colouration can be assessed by determining pigment dispersion in individual chromatophores or in multicellular chromatosomes (e.g. [19, 21]). The traditional and widely used CI [20] classifies individual chromatophores or chromatosomes based on their physiological state, indexing their extent of dispersion. As a result, the CI does not provide information on their morphological state (abundance of pigments). Animals with widely spaced, but fully dispersed, chromatosomes (Fig. 3, D) have, consequently, the same maximum index (CI = 5) as animals with a high abundance and overlap of chromatosomes (Fig. 3, H), even though the difference in darkness is visually apparent. This issue has already been considered by Parker [22] who observed catfish with clear differences in darkness, not distinguishable by the values of CI (all falling in the maximum category). Methods relying on the measurement of the diameter of the chromatosomes [19, 56] have the same problem, since they also omit morphological variation [22]. PiC combines both information on the distribution and abundance of pigments and is, therefore, able to distinguish physiological differences within the same animal (Fig. 3, A vs. E) and morphological differences between animals with the same physiological chromatosome state (Fig. 3, F vs. G), even in very dark animals (PiC > 80%). The comparison between PiC and CI shows the range where it is possible to transform the values from one method to the other and where PiC is more precise than CI. The logarithmic relationship indicates that the more dispersed the pigments are, the more effective the PiC is in detecting small differences between images compared to the CI. Thresholding methods are considered a more reliable tool for image analysis than human judgement [44]. Nevertheless, the accuracy and objectivity of PiC is influenced by the amount of manual adaptation applied. The database used during this study consisted of images taken under a variety of lighting conditions to show the wide applicability of PiC. However, automatic thresholding algorithms work best with images taken with identical lighting conditions and camera settings. Manual adaptation of the threshold values, required in cases where the image quality was not optimal (e.g. Fig. 3, B and C), resulted in increased observer variation and subjectivity. In studies where standardization of the images is not possible, extra care should be taken to ensure the objectivity of the study (e.g. observers being made blind to the treatments; between-observer repeatability analysis). These considerations should also be taken into account for the CI. The CI is furthermore less precise in darker animals, and it takes up to 4 times longer than PiC. This difference in analysis speed is due to the fact that the CI can only be determined by the manual classification of every single chromatosome in the image. Moreover, PiC allows testing for transparency, which is important in studies of colour change [21, 57].

Digital photography is a popular technique in animal colouration research due to its availability, speed, relative low price and ease of data acquisition [41, 58, 59]. Although there are issues with the use of digital images in animal colour studies [41], most of these relate to the control for variation in lightning conditions and the conversion of images to animal vision systems [41, 59]. Most cameras produce non-linear images (e.g. sRGB) that generally over- or underestimate light values and rigorous image analysis methods should include linearization and normalization of these images [41, 59]. PiC focusses on *a priori* specified pigments and does not rely on the exact colour or observer’s vision system. In this method, the difference between foreground and background pixels in an image is more important than the exact colour, thus stable lighting conditions are less relevant for PiC than for methods requiring linearized images. Studies that analyse chromatophores and pigment migration (see Table 1 for examples) usually focus on a limited number of pigments, in high contrast with the background. In these types of studies, PiC can be used also with sRGB images (as shown by the concordant PiC values of sRGB and linearized images reported above) as long as the users are aware of the limitations of the use of non-linear images. In cases where a more precise, objective and rigours determination of animal colour is required, image normalization and standardization can be performed prior to PiC determination. Standardization of lighting conditions and camera settings is also advised in these cases. Besides being less constrained regarding lighting conditions, PiC is also easy to use and fast in the analysis of large surfaces (opposed to spectrometry; [3]).

The study of animal colouration is a broad field of investigation encompassing molecular, cellular, physiological, behavioural and evolutionary questions [1, 12]. The proposed methodology combines the advantages of digital image acquisition with the power of a free open-source program. PiC is simple to use, can also be easily employed for educational purposes [60] and can be applied in any system where rapid colour change is determined by pigment migration in chromatophores. The brown shrimp’s chromatosomes system is a widely applicable model since its physiological factors are well studied and its pigment system is complex and essentially similar to those of vertebrates [8, 13, 61–63]. The proposed method will thus be a useful tool in future investigations on animal colouration as a fast and effective proxy for the interpretation of complex and dynamic biological systems in a wide range of species.

## Supplementary Material

Supplementary Data
