# Supplementary material for: Quantifying pigment cover to assess variation in animal colouration
Source: Biol Methods Protoc. 2017 Mar 27;2(1):bpx003. doi: 10.1093/biomethods/bpx003 (PMC6994029; doi:10.1093/biomethods/bpx003)
Supplement: Supplementary Data [file bpx003_supp.docx]

**Supporting information**

**Supporting figures**


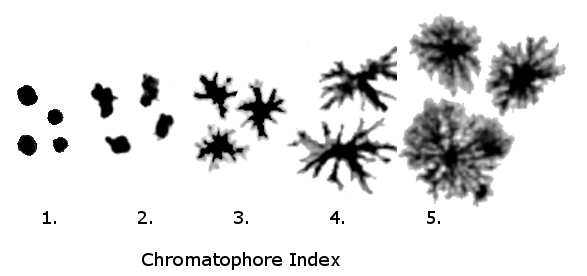


**Fig. S1. Stylised representation of the different classes of pigment dispersion in *Crangon crangon* chromatophores**. Classification is based on the Melanophore Index of Hogben and Slome (1931).

**
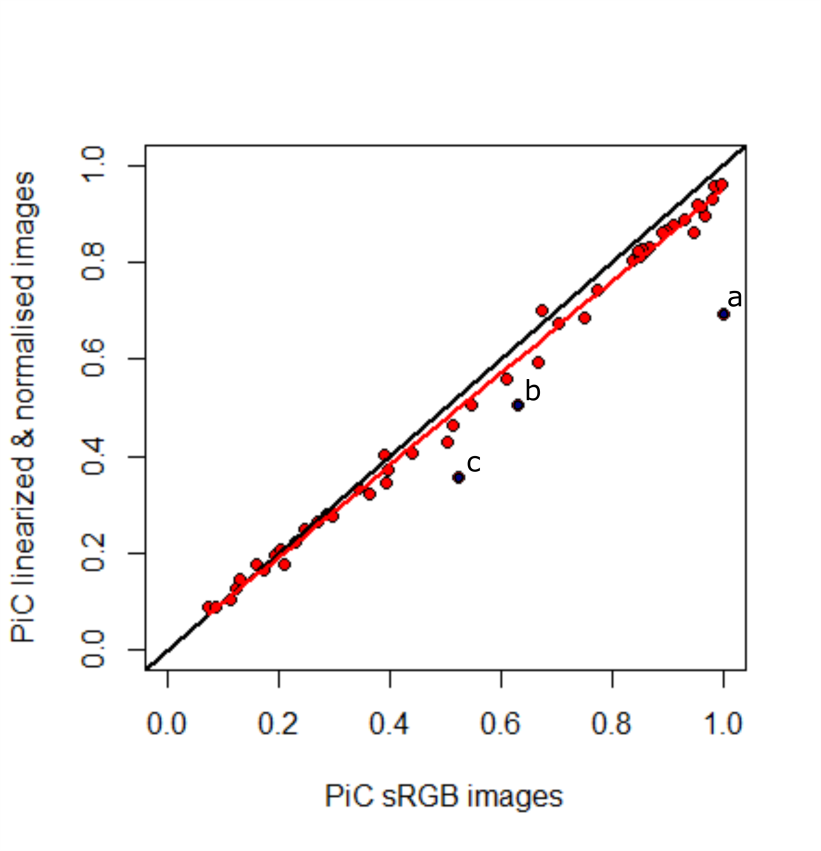
**

**Fig. S2. Relationship between the dark pigment cover fraction (PiC) with and without linearization and normalisation of 50 sRGB colour images of *Crangon crangon*’s exopods**. The red line shows the linear regression fit. Three outliers (a-c) were identified and removed prior to analysis, since the thresholding algorithm could not produce reliable PiC estimates without manual adaptation (due to image quality and exposure, as confirmed by visual observation).

**Supporting TABLES**

**Table S1.** **Regression parameters describing the relationship between chromatophore index and dark pigment cover fraction for three observers.**

| Observer | R^2^ | Intercept | Slope |
| --- | --- | --- | --- |
| 1 | 0.934 | -3.1625 | 0.61736 |
| 2 | 0.9125 | -3.3798 | 0.65672 |
| 3 | 0.9444 | -3.2235 | 0.63221 |
